# Violence risk assessment in psychiatric patients in China: A systematic review

**DOI:** 10.1177/0004867415585580

**Published:** 2016-01

**Authors:** Jiansong Zhou, Katrina Witt, Yutao Xiang, Xiaomin Zhu, Xiaoping Wang, Seena Fazel

**Affiliations:** 1 Mental Health Institute of The Second Xiangya Hospital, Hunan Province Technology Institute of Psychiatry, Key Laboratory of Psychiatry and Mental Health of Hunan Province, Central South University, Changsha, China; 2 Department of Psychiatry, Warneford Hospital, University of Oxford, Oxford, UK; 3 Unit of Psychiatry, Faculty of Health Sciences, University of Macau, Macau, China

**Keywords:** Violence, risk assessment, systematic review, prediction, China

## Abstract

**Objectives:**

The aim of this study was to undertake a systematic review on violence risk assessment instruments used for psychiatric patients in China.

**Methods:**

A systematic search was conducted from 1980 until 2014 to identify studies that used psychometric tools or structured instruments to assess aggression and violence risk. Information from primary studies was extracted, including demographic characteristics of the samples used, study design characteristics, and reliability and validity estimates.

**Results:**

A total of 30 primary studies were identified that investigated aggression or violence; 6 reported on tools assessing aggression while an additional 24 studies reported on structured instruments designed to predict violence. Although measures of reliability were typically good, estimates of predictive validity were mostly in the range of poor to moderate, with only 1 study finding good validity. These estimates were typically lower than that found in previous work for Western samples.

**Conclusion:**

There is currently little evidence to support the use of current violence risk assessment instruments in psychiatric patients in China. Developing more accurate and scalable approaches are research priorities.

## Introduction

Treatment practice guidelines in many Western countries recommend the assessment of violence risk in individuals with serious mental illness, particularly schizophrenia (American Psychiatric Association, 2004; McGorry et al., 2005; National Institute for Health and Clinical Excellence, 2009). Until late 2012, however, there were no national mental health laws in China and no legislation to mandate the assessment of violence risk in those with a serious mental illness. Article 30 of the new National Mental Health Law, however, provides for the involuntary commitment of mentally disordered persons providing that two conditions are met: (1) the individual is diagnosed with a serious mental illness and (2) the individual poses a risk to either self or others (Shao and Xie, 2013). Both these criteria must be satisfied through a diagnostic and risk assessment (Zhao and Dawson, 2014). Survey data suggest that China has an estimated 173 million psychiatric patients (Phillips et al., 2009), and 728 hospitals as of 2012 (*Chinese Health Statistics Yearbook*, 2013). The introduction of this new law will therefore have widespread implications.

Traditionally, mental health professionals in China have tended to rely on unstructured clinical judgment when assessing violence risk in psychiatric patients (Ho et al., 2013). In many Western countries, however, structured assessment instruments are commonly used in both forensic and general psychiatric units for violence risk assessment (Archer et al., 2006; Higgins et al., 2005; Khiroya et al., 2009). Although these tools are rarely used as the sole basis for clinical decision-making owing to their low positive predictive values (PPVs) (Ryan et al., 2010), the way in which the dangerousness criterion is to be operationalized under China’s new mental health law is, at present, unclear (Shao and Xie, 2013), leaving the decision as to how to satisfy this requirement open to the discretion of those undertaking the assessment (Ding, 2014). Determining violence risk from structured clinical judgment (SCJ) tools may represent one approach that mental health professionals in China may adopt to satisfy this criterion. More likely, though, these tools are being introduced as part of a range of measures to improve patient care, and identifying high-risk groups could enable targeted interventions to be introduced and resources to be directed toward those at highest risk of adverse outcomes.

These instruments, however, have mostly been developed and validated in Western samples. Given that China’s culture, legislation and psychiatry services are different, it has been argued that these violence risk assessment instruments may be associated with lower predictive validity when used in Chinese psychiatric populations (Yao et al., 2014b). A recent review concluded that some SCJ tools provide high levels of reliability and validity in Chinese samples, particularly the Chinese version of the Historical, Clinical, Risk Management–20 (HCR-20) and the Violence Risk Screening–10 (V-RISK-10) (Gu et al., 2014). However, this review was limited in four ways: (1) it focused on mentally disordered offenders rather than general psychiatric patients and offender populations, (2) it did not consider three popular tools currently used to assess violence risk in China (i.e. the Violence Risk Scale–Chinese version [VRS-C], the Psychopathy Checklist–Revised [PCL-R] and the Brøset Violence Checklist [BVC]), (3) it did not compare the predictive validity of Chinese-developed instruments to Western-developed ones and (4) the review lacked clear inclusion and exclusion criteria.

We have therefore conducted a systematic review of the use of risk assessment instruments for the prediction of violence to synthesize the evidence base for the reliability and validity of such tools in Chinese samples. Our aim was to examine three main areas: (1) the current state of risk assessment research in China, (2) the instrument most frequently used to assess aggression and violence risk in China and finally (3) whether these instruments are associated with a similar degree of predictive validity as found in Western samples.

## Methods

### Search strategy

Eight computerized databases were searched for studies published between 1 January 1980 and 3 June 2014: Medline, EMBASE, PsycINFO, the Chinese Journal Full-text Database (CJFD), the Chinese Biomedical Literature Database (CBM), National Science and Technology Library (NST), WANFANG data and the Database Research Center of the Chongqing Branch of the Institute of Scientific & Technical Information of China (CB-ISTIC). Combinations of the following keywords were used to identify relevant studies: *aggression OR violence OR psychopathy AND risk assessment OR prediction.* Reference lists were also hand-searched to identify additional studies.

### Inclusion and exclusion criteria

Studies were eligible for inclusion if they were conducted in mainland China and examined the reliability and/or validity of a psychometric tool or risk assessment instrument designed to assess or predict the likelihood of either aggression or violence. Although previous work suggests that the inclusion of studies based on the original calibration sample will lead to effect size inflation (Blair et al., 2008), we nevertheless included such studies as we wished to provide an overview of all instruments, including locally developed instruments, currently used in psychiatric practice in China.

Studies that used violence risk assessment instruments to estimate the prevalence of violence, but did not report data on the reliability or predictive validity of these instruments were excluded (Chen and Zhou, 2012). Where multiple publications used overlapping samples, we included only the study with the largest sample size to avoid double-counting.

### Data extraction

Data were extracted by two researchers working independently (J.Z. and X.Z.) using a standardized form, which included information on demographic and descriptive features of the sample, and reliability and validity statistics from each study. Measures of reliability included Cronbach’s alpha, the intraclass correlation coefficient (ICC), test–retest reliability, split-half reliability and the inter-rater consistency coefficient. Measures of validity included the area under the receiver operating characteristic curve (AUC), sensitivity, specificity and positive and negative predictive values (PPVs and NPVs). No one measure of reliability or validity was preferred; rather, a combination of statistics should be examined as part of any judgment about the performance of any tool. Additionally, for locally developed tools, information on item content was also extracted. If there were any uncertainties, these were clarified in consultation with one of the co-authors (K.W.).

## Results

### Characteristics of the included studies

**Figure 1.**
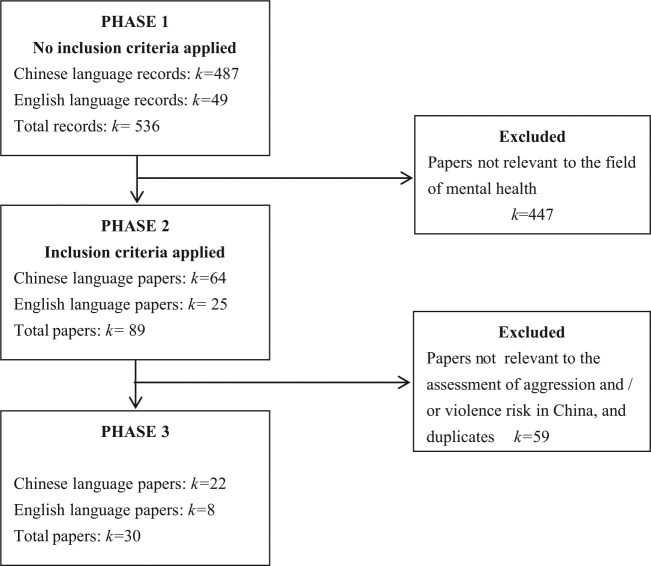
Systematic review search strategy flow diagram.

The initial search identified a total of 528 records including 481 in Chinese and 47 in English. Another 8 records (6 in Chinese and 2 in English) were identified after searching reference lists of other reviews. Following application of the inclusion criteria, the number of potentially eligible records was reduced to 89 (64 in Chinese and 25 in English). When exclusion criteria were applied, the final number of records included in this review was reduced to 30 (22 in Chinese and 8 in English) (Figure 1). Studies were most commonly excluded because they were not concerned with the assessment of violence risk.

### Tools for aggression assessment

Six of the 30 primary studies assessed the reliability and validity of tools measuring aggression (Table 1). The instruments used for the assessment of aggression were the Modified Overt Aggression Scale (MOAS; *k* = 5; 83%) and a locally developed instrument (*k* = 1; 17%). Half of these studies were conducted in mixed adult forensic and general psychiatric samples (*k* = 3).

**Table 1.** An overview of tools assessing aggression in China.

Reference	Tool/s	Approach	*n*	Age	Sex	Study setting	Study design	Aggression risk factors
Chen and Deng (2012)	Self-developed instrument	Actuarial	1465	Violent group: 35.8 ± 11.5 years. Nonviolent group: 35.1 ± 11.3 years.	Males: 100%.	General psychiatric hospital.	Prospective	Delusions, hallucinations, mood state, treatment adherence and substance abuse.
Shao et al. (2010)	SSP and MOAS	Actuarial	400	Overall sample: 15–18 years.	Males: 100%.	Youth detention center.	Retrospective	Impulsiveness, trait irritability, verbal trait aggression and physical trait aggression.
Wang (2012)	MOAS, PANSS and TPQ	Actuarial	122	Violent group: 40.7 ± 9.7 years. Nonviolent group: 48.5 ± 11.4 years.	Males: 100%.	Forensic and general psychiatric hospitals.	Cross-sectional	Novelty seeking and reward dependence.
Yang (2007)	MMPI, MOAS	Actuarial	101	Overall sample: 18–50 years.	Males: 100%.	Prison.	Retrospective	Unemployment, young age, previous violence, impulsiveness, lack of social supports, experienced childhood abuse, lack of regret, mental state and substance abuse.
Zhang and Hu (2011)	MOAS	Actuarial	490	Violent group: 36 ± 12 years. Nonviolent group: 39 ± 18 years.	Males: 40.6% (*n* = 199). Females: 31.8% (*n* = 156). Unreported: 27.1% (*n* = 133).	Forensic and general psychiatric hospitals.	Retrospective	
Zhuang et al. (2006)	MOAS	Actuarial	78	Violent group: 36.2 ± 11.5 years. Nonviolent group: 38.6 ± 10.1 years.	Males: 67.9% (*n =* 53). Females: 32.1% (*n =* 25).	Forensic and general psychiatric units.	Retrospective	Positive psychotic symptoms, depression or paranoid personality disorder, past violence and alcohol abuse history, early aversive family environment.

SSP: Swedish University Scales of Personality; MOAS: Modified Overt Aggression Scale; PANSS: Positive and Negative Symptom Scale; TPQ: Tridimensional Personality Questionnaire; MMPI: Minnesota Multiphasic Personality Inventory.

### Tools for violence risk assessment and prediction

The remaining 24 primary studies reported information for a violence risk assessment tool (Table 2). Ten reports (42%) employed a locally developed violence risk assessment instrument, another 14 (60%) used tools developed in Western countries. These included the Violence Risk Scale (VRS; *k* = 4; 17%), the HCR-20 (*k* = 3; 12%), the PCL-R (*k* = 2; 8%), the V-RISK-10 (*k* = 3; 12%), the BVC (*k* = 2; 8%), the Structured Assessment of Violence Risk in Youth (SAVRY; *k* = 1; 4%), the Level of Service Inventory–Revised (LSI-R; *k* = 1; 4%) and a Chinese modified version of the Violence Scale (VS-CM; *k* = 1; 4%). The majority of these studies were conducted in adult general psychiatric cohorts (*k* = 11; 44%).

**Table 2.** Study characteristics, reliability and validity information for violence risk assessment tools in China.

Reference	Tool/s	Approach	*n*	Age	Sex	Study design	Study setting	Inpatient/ outpatient	Domains assessed	Reliability	Validity
Chan (2014)	CRAT-P	Actuarial	2225	18 years or above.	Male: 100%.	Retrospective	Community		Static, dynamic		**AUC**: 0.76. **Sensitivity**: 61.0%. **Specificity**: 64.2%.
Chen et al. (2014)	VS-CM	Actuarial	107	33.4 ± 11.9 years.	Male: 30.8% (*n* = 33). Female: 69.2% (*n* = 74).	Prospective	Acute psychiatric ward	Inpatients	Static, dynamic		**AUC**: 0.80. **Sensitivity**: 97.0%. **Specificity**: 35.0%.
Deng et al. (2008)	SD	Actuarial	1440	35.0 ± 10.9 years.	Males: 66.3% (*n* = 955). Females: 33.7% (*n* = 485).	Prospective	General psychiatry hospital	Inpatients	Static, dynamic		
Han and Zhao (2013)	SD	Actuarial	397	15–40 years.	Males: 55.2% (*n* = 219). Females: 44.8% (*n* = 178).	Prospective	General psychiatric hospital	Inpatients	Static, dynamic		
Ho et al. (2013)	HCR-20	SCJ	220	19–78 years.	Males: 75.0% (*n* = 165). Females: 25.0% (*n* = 55).	Prospective and retrospective	General and forensic psychiatric hospital	Outpatients	Static, dynamic	**ICC**: H subscale score: 0.71. C subscale score: 0.43. R subscale score: 0.37. Total score: 0.73.	**AUC**: Total score (6 months): 0.70. Total score (12 months): 0.67.
Wang et al. (2009)	SAVRY	SCJ	109	Violent group: 16.1 ± 1.3 years. Nonviolent group: 16.1 ± 1.2 years.	Males: 100%.	Retrospective	Youth Detention Centers (YDCs)		Static		
Li et al. (2010)	SD	SCJ	860	47.9 ± 14.3 years.	Males: 47.3% (*n* = 407). Females: 52.7% (*n* = 453).	Retrospective	Community	Outpatients	Static	**Cronbach’s alpha**: 0.86.	
Liu et al. (2010b)	SD	Actuarial	878	Offenders: 23.6 ± 1.7 years. Controls:23.6 ± 1.7 years.	Males: 100%.	Prospective	Prison		Static	**Cronbach’s alpha**: Violence outcome: 0.78. Antisocial behavior outcome: 0.74. Anger outcome: 0.89. Violent attitude outcome: 0.83. **Test–retest reliability**: Violence outcome: 0.80. Antisocial behavior outcome: 0.83. Anger outcome: 0.80. Violent attitude outcome: 0.77.	
Liu et al. (2010a)	PCL-R	Actuarial	60	20–38 years.	Males: 100%.	Retrospective	Prison		Static	**ICC**: 0.81. **Cronbach’s alpha**: 0.85.	**Correlation coefficient**: Between PCL-R and PDQ: 0.59.
Lv et al. (2013)	HCR-20	SCJ	156	33.8 ± 11.3 years.	Males: 100%.	Prospective + retrospective	General psychiatric hospital	Inpatients	Static, dynamic	**Cronbach’s alpha**: Historical subscale: 0.62. Clinical subscale: 0.58. Risk management subscale: 0.66. Total score: 0.78. **Intraclass correlation coefficient**: Historical subscale: 0.97. Clinical subscale: 0.92. Risk management subscale: 0.82. Total score: 0.85. **Test–retest reliability**: Historical subscale: 0.95. Clinical subscale: 0.52. Risk management subscale: 0.77. Total score: 0.88.	**AUC**: Historical subscale: 0.73. Clinical subscale: 0.63. Risk management subscale: 0.60. Total score: 0.72. **PPV**: 60.0%. **NPV**: 77.3%. **Percent correctly classified**: 75.6%.
Shi et al. (2012)	PCL-R	Actuarial	109	31.6 ± 12.1 years.	Males: 76.1% (*n* = 83). Females: 23.9% (*n* = 26).	Retrospective	Forensic psychiatry hospital	Inpatients	Static		
Shi et al. (2014)	BVC, V-RISK-10	Actuarial	118	35.6+13.5 years.	Males: 57.6% (*n* = 68). Females: 42.4% (*n* = 50).	Prospective	General psychiatric hospital	Inpatients	Static, dynamic	**BVC** **ICC**: 0.95. **Cronbach’s alphas**: 0.81. **V-RISK-10** **ICC**: 0.91. **Cronbach’s alphas**: 0.69.	**BVC** **AUC**: 0.79. **Sensitivity/specificity**: 96.3%/60.9%. **PPV**: 67.5%. **V-RISK-10** **AUC**: 0.72. **Sensitivity/specificity**: 87.0%/57.8%. **PPV**: 63.5%.
Tian (2013)	SD	Actuarial	57	34.8 ± 5.9 years.	Males: 61.4% (*n* = 35). Females: 38.6% (*n* = 22).	Prospective	General psychiatric hospital	Inpatients	Static, dynamic		
Wang (2012)	SD	Actuarial	6633	15.1 ± 0.9 years.	Boys: 49.4% (*n* = 3280). Girls: 50.6% (*n* = 3353).	Retrospective	School		Static	**Cronbach’s alphas**: Boys: 0.92, girls: 0.89. **Test–retest reliability**: 0.76.	**Correlation coefficient**: Between the instrument and aggressive behavior subscales of YSR: 0.54. Between the instrument and rule-breaking behavior subscales of YSR: 0.68.
Wang (2012)	VRS	Actuarial	501	41 ± 15 years.	Males: 49.7% (*n* = 249). Females: 50.3% (*n* = 252).	Retrospective	Community		Static		
Wei and Ma (2013)	SD	Actuarial	148	18–54 years.	Males: 100%.	Prospective	General psychiatric hospital	Inpatients	Static, dynamic		
Xiao et al. (2010)	HCR-20	SCJ	60	Violent group: 31.4 ± 9.4 years. Nonviolent group: 31.1 ± 10.1 years.	Males: 100%.	Retrospective	Forensic and general psychiatry hospital	Outpatients	Static, dynamic	**Cronbach’s alphas**: 0.92. **Test–retest reliability**: 0.90.	**Correlation coefficient**: Between HCR-20 and MOAS: 0.84.
Yang and Zhao (2011)	SD	SCJ	10	15–23 years.	Males: 50.0% (*n* = 5). Females: 50.0% (*n* = 5).	Retrospective	YDC		Static		
Yao et al. (2012)	VRS	Actuarial	376	34.7 ± 12.5 years.	Males: 46.5% (*n* = 175). Females: 53.5% (*n* = 201).	Prospective	General psychiatric hospital	Inpatients	Static, dynamic	**ICC**: 0.89.	**AUC**: 0.63. **Sensitivity/specificity**: 0.80/0.38. **PPV**: 34%. **NPV**: 82%.
Yao et al. (2014a)	BVC	Actuarial	281	34.5 ± 11.6 years.	Males: 43.1% (*n* = 121). Females: 56.9% (*n* = 160).	Prospective	General psychiatric hospital	Inpatients	Static, dynamic		**AUC**: 0.85. **Sensitivity/specificity**: 78.5%/ 88.2%.
Yao et al. (2014b)	VRS	Actuarial	397	34.1 ± 12.4 years.	Males: 56.9% (*n* = 226). Females: 43.1% (*n* = 171).	Prospective	General psychiatric hospital	Outpatients	Static, dynamic		**AUC**: 0.62. **Sensitivity/specificity**: 79.2%/33.3%. **PPV**: 9.9%. **NPV**: 94.5%.
Zhan et al. (2013)	V-RISK-10	Actuarial	109	33.1 ± 11.2 years.	Males: 60.6% (*n* = 66). Females: 39.4% (*n* = 43).	Prospective	General psychiatric hospital	Inpatients and outpatients	Static	**Cronbach’s alphas**: 0.83.	**Correlation coefficient**: Between each of the 10 items with the MOAS total score were from 0.30 to 0.59.
Zhang et al. (2012)	VRS	Actuarial	125	32.5 ± 10.9 years.	Males: 76.0% (*n* = 95). Females: 24.0% (*n* = 30).	Retrospective	Forensic psychiatry hospital	Inpatients	Static	**ICC**: 0.80. **Cronbach’s alpha**: 0.92. Split-half reliability: 0.91.	
Zhang and Liu (2014)	LSI-R	Actuarial	305	31.3 ± 12.9 years.	Males: 88.2% (*n* = 269). Females: 11.8% (*n* = 36).	Retrospective, prospective	Community		Static, dynamic	**Cronbach’s alpha**: LSI-R total: 0.85. Criminal history: 0.75. Education/Employment: 0.78. Finance: 0.12. Family/Marital: 0.68. Accommodation: 0.66. Leisure/Recreation: 0.62. Companions: 0.80. Alcohol/Drug problems: 0.77. Emotional/Personal problems: 0.21. Attitudes/Orientations: 0.77.	

SCJ: structured clinical judgment instrument; HCR-20: Historical, Clinical, Risk Management–20; SD: self-developed tool; SAVRY: Structured Assessment of Violence Risk in Youth; VRS: Violence Risk Scale; V-RISK-10: Violence Risk Screening–10; YSR: Youth Self-Report Form; PCL-R: Psychopathy Checklist–Revised; CRAT-P: Chinese Risk Assessment Tool for Perpetrators; BVC: Brøset Violence Checklist; VS-CM: Chinese modified version of the Violence Scale; PDQ: Personality Diagnostic Questionnaire; ICC: Intraclass correlation coefficient; AUC: Area Under the receiver operating characteristic (ROC) Curve; PPV: Positive Predictive Value; NPV: Negative Predictive Value; LSI-R: Level of Service Inventory–Revised; MOAS: Modified Overt Aggression Scale.

### Reliability and validity of tools for the assessment of aggression

None of the six included studies of aggression tools reported information on reliability or validity. Rather, they all investigated risk factors associated with aggression. Substance abuse was most commonly identified as a significant risk factor for aggression in these studies (*k* = 3; 50%), followed by a previous history of aggression and/or violence (*k* = 3; 50%), positive symptomatology (*k* = 2; 33%) and impulsiveness (*k* = 3; 33%). Demographic factors, such as young age, unemployment and early adverse experiences, were also described as risk factors in three studies.

### Reliability and validity of tools for the assessment of violence risk

Of the 24 included studies, 15 (63%) reported information on reliability, which was assessed using the following statistics: Cronbach’s alpha, the ICC, test–retest reliability, split-half reliability and the inter-rater consistency coefficient. Most of the locally developed instruments did not report reliability and validity statistics. A summary of these statistics is provided in Table 3.

**Table 3.** Summary of the reliability and validity statistics for Western-developed violence risk assessment instruments used in China.

Statistic	Reference	Poor/small	Acceptable/moderate	Good/fair	Excellent
Reliability					
Cronbach’s alpha^a^		0.5 ⩾ α < 0.6	0.6 ⩾ α < 0.7	0.7 ⩾ α < 0.9	α ⩾ 0.9
	Shi et al. (2014)		V-RISK-10	BVC	
	Liu et al. (2010a)			PCL-R	
	Lv et al. (2013)			HCR-20	
	Zhan (2013)			V-RISK-10	
	Zhang and Liu (2014)			LSI-R	
	Zhang et al. (2012)				VRS
	Xiao et al. (2010)				HCR-20
ICC^b^		⩽0.40	0.41 ⩾ ICC ⩽ 0.60	0.61 ⩾ ICC ⩽ 0.80	0.81 ⩾ ICC ⩽ 1.00
	Zhang et al. (2012)			VRS	
	Ho et al. (2013)			HCR-20	
	Yao (2014b)				V-RISK-10
	Liu et al. (2010a)				PCL-R
	Yao et al. (2012)				VRS
	Shi et al. (2014)				BVC
					V-RISK-10
Test–retest reliability				⩾0.70	
	Xiao et al. (2010)			HCR-20	
Validity					
AUC^c^		0.60 ⩾ AUC > 0.70	0.70 ⩾ AUC > 0.80	0.80 ⩾ AUC > 0.90	AUC ⩾ 0.90
	Yao (2014b)	V-RISK-10			
	Yao et al. (2014b)				
	Shi et al. (2014) Lv et al. (2013)		BVC HCR-20		
	Shi et al. (2014)		V-RISK-10		
	Ho et al. (2013)	HCR-20 (12 months)	HCR-20 (6 months)		
	Chan (2014)		CRAT-P		
	Chen et al. (2014)			VS-CM	
	Yao et al. (2014a)			BVC	

V-RISK-10: Violence Risk Screening–10; BVC: Brøset Violence Checklist; PCL-R: Psychopathy Checklist–Revised; HCR-20: Historical, Clinical, Risk Management–20; LSI-R: Level of Service Inventory–Revised; VRS: Violence Risk Scale; ICC: Intraclass correlation coefficient; AUC: Area Under the receiver operating characteristic (ROC) Curve; CRAT-P: Chinese Risk Assessment Instrument for Perpetrators; VS-CM: Chinese modified version of the Violence Scale.

References for the interpretive cut-points for the reliability and validity statistics used in this table:

a Kline (2000).

b Landis and Koch (1977).

c Swets (1988).

Using Cronbach’s alpha, there was evidence of good reliability for five instruments: the BVC, PCL-R, HCR-20, V-RISK-10 and the LSI-R, and excellent reliability for two instruments: the VRS and HCR-20. According to the ICC, there was evidence of good reliability for the VRS and HCR-20, and excellent reliability for the V-RISK-10, the PCL-R, the VRS and the BVC. Only one study using the HCR-20 reported the test–retest reliability.

Information on validity was reported in 12 studies (50%) using the following statistics: AUC, sensitivity and specificity and positive and negative predictive values. Validity statistics are also summarized in Table 3. Using the AUC, there was evidence of poor validity for the V-RISK-10, the VRS and the HCR-20 over a 12-month follow-up period. There was evidence of moderate validity for the BVC, V-RISK-10, the HCR-20 over a 6-month follow-up period and the CRAT-P.

## Discussion

As China invests more into mental health care, increasing attention will be paid to reducing adverse outcomes in patient groups. One approach that this has taken in many countries is to introduce the routine use of violence risk assessment instruments to assist in identifying high-risk groups and manage violence risk more actively. In addition, the 2012 new National Mental Health Law may also increase the use of such instruments as an aid to clinical decision-making regarding involuntary treatment in hospital. This systematic review therefore investigated the reliability and validity of structured violence risk assessment instruments in China. A total of 15 risk assessment tools were identified, 7 involving instruments originally calibrated and validated in Western samples and 8 developed in Chinese populations. Data on both reliability and validity of these instruments were extracted from 24 studies involving 15,681 participants. Results of this review have three main implications for research into the assessment of violence risk in China and clinical practice.

First, although Western-developed instruments, such as the HCR-20, demonstrated good reliability in this review, predictive validity estimates were often noticeably lower than those found in Western samples (Singh et al., 2011), suggesting there is little evidence to support the use of current instruments for the prediction of future violence risk in China at present. The lower predictive validity of these instruments observed in this review is particularly important as it suggests that these instruments should not be used as sole determinants for eligibility for involuntary detention under Article 30 of China’s new Mental Health Law or for other medico-legal decisions in patients.

The lower predictive validity of existing instruments may stem from the inclusion of items within these violence risk assessment schemes that have little salience for the prediction of risk in Chinese samples. Work, for example, suggests that Asian Americans score significantly lower on a number of the historical items on the HCR-20 as compared to Caucasian patients. Instead, violence in Asian American psychiatric patients was more strongly associated with scores on the clinical subscale of the HCR-20 (Fujii et al., 2005). Further work suggests that the AUC of established violence risk assessment instruments cannot distinguish between violent and nonviolent offenders at greater than chance levels for those patients of Middle Eastern descent (Långström, 2004). The improvement of violence risk assessment in China may therefore benefit from the development of evidence-based instruments based on local research. Furthermore, the sheer scale of psychiatric patient numbers in China suggests that scalable instruments need to be developed, rather than those that require external training, take considerable time to implement and require money to use.

A number of investigations included in the review assessed validity using correlation coefficients against tools that assess aggression or psychopathy. These are of limited interest as the violence risk assessment tools considered in this review are intended to be used to predict more serious outcomes. Most included studies investigated predictive validity using the AUC. Predictive validity, however, can be broken down into two components: discrimination and calibration. The AUC, however, captures only discrimination. Given that a goal of violence risk assessment is to correctly stratify individuals into risk categories, the calibration ability of a risk assessment instrument is arguably of greater concern (Cook, 2007). As there are presently no guidelines as to how to combine aspects of discrimination and calibration (Witt et al., 2015), the assessment of predictive validity should employ statistics that adequately capture both discrimination and calibration (Singh, 2013). Recent work, for example, suggests that, at the very least, information on a combination of predictive validity estimates, including: PPVs and NPVs, sensitivity and specificity, number needed to detain (NNDs) and number safely released (NSRs) should be reported (Fazel et al., 2012). PPVs represent the proportion of patients predicted by an instrument to be at risk of violence who ultimately do commit a violent act while NPVs indicate the proportion judged at low risk of violence who do not commit a violent act (Singh, 2013). Greater adherence to existing guidelines for the reporting of clinical risk prediction research may also help to improve the reliability and applicability of work in this area (Bouwmeester et al., 2012).

Finally, we were unable to undertake a meta-analytic summary of the predictive validity of these instruments as the information required to calculate pooled AUCs was not routinely reported in the studies included in this review. While this approach may allow for comparison with the performance of these tools in Western samples, our focus in this paper was to evaluate the extent to which these tools could be used as a basis to justify involuntary treatment under China’s new Mental Health Law and for clinical decision-making in Chinese settings. A comparison of the predictive performance of these instruments between countries is beyond the scope of this paper.

### Conclusion

Although there are a large number of violence risk assessment instruments that are currently available to assist in the prediction of violence risk, these have almost entirely been developed and validated in Western samples. Presently, there is little evidence to support the use of these Western-developed violence risk assessment instruments in China. The assessment of violence risk in this population should be sensitive to a range of factors, including ease of use, cost and possibly risk factors unique to Chinese populations. Therefore, the development of more accurate and scalable approaches should improve the assessment of violence risk in psychiatric patients in China, and are urgently required.
